# Myc Prevents Apoptosis and Enhances Endoreduplication Induced by Paclitaxel

**DOI:** 10.1371/journal.pone.0005442

**Published:** 2009-05-06

**Authors:** Giuliana Gatti, Giovanna Maresca, Manuela Natoli, Fulvio Florenzano, Angelo Nicolin, Armando Felsani, Igea D'Agnano

**Affiliations:** 1 Department of Pharmacology, University of Milan, Milan, Italy; 2 CNR, Institute of Neurobiology and Molecular Medicine, European Brain Research Institute, S. Lucia Foundation, Rome, Italy; 3 Genomnia, Milan, Italy; Karolinska Institutet, Sweden

## Abstract

**Background:**

The role of the *MYC* oncogene in the apoptotic pathways is not fully understood. *MYC* has been reported to protect cells from apoptosis activation but also to sensitize cells to apoptotic stimuli. We have previously demonstrated that the down-regulation of Myc protein activates apoptosis in melanoma cells and increases the susceptibility of cells to various antitumoral treatments. Beyond the well-known role in the G1→S transition, *MYC* is also involved in the G2-M cell cycle phases regulation.

**Methodology/Principal Findings:**

In this study we have investigated how *MYC* could influence cell survival signalling during G2 and M phases. We used the microtubules damaging agent paclitaxel (PTX), to arrest the cells in the M phase, in a p53 mutated melanoma cell line with modulated Myc level and activity. An overexpression of Myc protein is able to increase endoreduplication favoring the survival of cells exposed to antimitotic poisoning. The PTX-induced endoreduplication is associated in Myc overexpressing cells with a reduced expression of MAD2, essential component of the molecular core of the spindle assembly checkpoint (SAC), indicating an impairment of this checkpoint. In addition, for the first time we have localized Myc protein at the spindle poles (centrosomes) during pro-metaphase in different cell lines.

**Conclusions:**

The presence of Myc at the poles during the prometaphase could be necessary for the Myc-mediated attenuation of the SAC and the subsequent induction of endoreduplication. In addition, our data strongly suggest that the use of taxane in antitumor therapeutic strategies should be rationally based on the molecular profile of the individual tumor by specifically analyzing Myc expression levels.

## Introduction

The *MYC* oncogene is an extensively studied gene, implicated in various cellular processes including growth, proliferation, loss of differentiation and cell death. It has been widely ascertained that *MYC* gene encodes a transcription factor, which trans-activates many genes involved in the regulation of the cell cycle progression [1]–[4]. Although many reported on involvement of *MYC* in the apoptotic pathways, the mechanism of its action is not yet fully understood [5]–[8]. The ability of Myc to trigger apoptosis may represent a key to the control of tumor development. Apoptosis might be an intrinsic safety mechanism to limit the life of a cell with upregulated *MYC* expression, thus preventing further transformation. However, loss of this function through any additional mutation that prevents Myc from promoting apoptotic events will impact on cell survival. It strongly correlates with Myc expression to allow continued proliferation, mutation and cancer evolution of the affected clones. Even though under appropriate conditions Myc overexpression can lead to apoptosis [5], [6], [9], also the down-regulation of Myc can sensitize the cells to the induction of apoptosis [10]–[14]. Our group as well have previously demonstrated that the Myc down-regulation is able to activate apoptosis and to sensitize melanoma cells to different antineoplastic agents [15]–[17].

The activation of different cell cycle checkpoints in the various cell cycle compartments functions as mechanisms of cellular surveillance and protects cells from genomic instability and deleterious effects induced by genotoxic agents [18], [19]. *MYC* overexpression influences cell cycle checkpoints, allowing some tumor cells to survive drug treatment and favoring the selection of a subpopulation with increased genomic instability and drug resistance or with a more aggressive tumor phenotype [5]. This effect has been attributed to the ability of *MYC* to induce DNA damage, promote gross chromosomal rearrangements, induce inappropriate cell cycle progression and impair DNA repair [20]. An alternative mechanism by which Myc can regulate proliferation in normal cells and cause genomic instability in tumors has been suggested by Dalla Favera group in that Myc controls DNA replication in a non-transcriptional way [21]. Myc has also been recently recognized as an important regulator of “stemness” as it is able both to activate an embryonic stem cell-like transcriptional module and, when ectopically expressed, to increase the cancer stem cell fraction and enhance tumorigenicity [22], [23]. In addition, when Myc overexpressing cells are exposed to mitotic spindle poisoning, rather than resulting in the typical G2-M arrest seen in control cells, an aberrant round of DNA synthesis, without an intervening mitosis (endoreduplication), occurs producing an aneuploid state [24]. However, the exact mechanism through which Myc mediates this response is only partially characterized.

Normal human fibroblasts stimulated with *MYC* traverse the G1 and S cell cycle phases and then arrest in the G2 phase becoming frequently aneuploid probably because of endoreduplication [25]. It has been characterized a Myc domain which regulates G2 arrest [26]. Some checkpoints monitor proper assembly of the mitotic spindle and prevent abnormal segregation of chromosomes during the metaphase to anaphase transition [27]. Myc oncoprotein attenuates the spindle assembly checkpoint (SAC) specifically activated by microtubules inhibitors and induces polyploid cells [28]. In addition, in HeLa cells α-tubulin molecules bound Myc protein, probably functioning as reservoir to sequester and release the Myc protein [29].

The aim of this study was to investigate the potential role of Myc in the survival signalling during G2 and M phases. We hypothesized that by reducing Myc expression the cells may not progress through M phase, arresting in the G2 phase and may activate the apoptotic program. We used the taxane paclitaxel (PTX) to block cells in the M phase and examined how the modulation of Myc could influence activation of apoptotic pathways in the G2 and M phases.

In order to distinguish the effective role of Myc from that exerted by p53 in microtubules damaged cells we chose a cellular model in which p53 is mutated and therefore unable to be activated after damage. We showed that Myc overexpression is able to reduce apoptosis and enhance endoreduplication induced by PTX in the p53-mutated M14 melanoma cell line. In addition, we localize Myc protein at the centrosomes during prometaphase. The presence of Myc at the spindle poles during the prometaphase could be necessary for the Myc-mediated attenuation of the SAC and the subsequent induction of endoreduplication.

## Materials and Methods

### Cell cultures and transfections

M14 human melanoma cell line (control cells) was cultured in RPMI-1640 medium supplemented with 10% fetal calf serum (Hyclone), L-glutamine (1%) and antibiotics at 37°C in a 5% CO_2_/95% air atmosphere in a humidified incubator.

M14 cell clones stably expressing an ecdysone-inducible *MYC* antisense mRNA were maintained in RPMI-1640, as above, in the presence of both G418 (GIBCO) and Zeocyn (Invitrogen) [16]. Ponasterone A (Invitrogen) was used to induce the *MYC* AS RNA transcription in pIND*c-myc* AS clone, with a dose of 20 µM every 24 h. The Myc down-regulated cells are called Myc^(−)^ cells throughout the paper.

Generation of stable *MYC* overexpressing cell clones (called Myc^(+)^ throughout the paper) was achieved by transfecting the control M14 cell line with the pCDNA3-hMyc plasmid expressing the full-length *MYC* coding sequence driven by the CMV promoter. Forty-eight hours after transfection, the cells were selected for 3 weeks in the presence of G418 (800 µg/mL).

C2C12 (American Type Culture Collection, CRL 1772) and C2C12-Myc [30] cells were propagated in proliferation Dulbecco's Modified Eagle's Medium (DMEM, GIBCO) with 4,5 g/ml of glucose, supplemented with 20% fetal calf serum (Hyclone), 20 mmol L-glutamine and antibiotics. The cultures were grown at 37°C under a humidified atmosphere of air with 5% CO_2_.

Caco2 cells (INSERM, Villejuif, France) were subcultured at 50% density in a 90% air/10% CO_2_ atmosphere in DMEM containing 25 mM glucose, 3.7 g/L NaHCO_3_ and supplemented with 4 mM L-glutamine, 1% non essential amino acids, antibiotics, and 10% heat-inactivated foetal calf serum (Hyclone).

HeLa cells were subcultured in a 95% air/5% CO_2_ atmosphere in DMEM containing 25 mM glucose, 3.7 g/L NaHCO_3_ and supplemented with 2 mM L-glutamine, antibiotics, and 10% heat-inactivated foetal calf serum (Hyclone).

### Treatments

To arrest the cells in the mitotic compartment we used paclitaxel (PTX). 1×10^5^ cells for each M14 cell line were seeded in 12-wells plates. Twenty-four hours after seeding, the *MYC* AS clone was induced with ponasterone; and after further 24 h all the three M14 cell lines were exposed to PTX for 24 and 48 h. At each time-point, cells were harvested and counted by using the trypan blue dye exclusion test.

C2C12 cells, diluted (1∶13) and plated the day before, were treated for 15 h with PTX 100 nM. Cells were harvested, pooled with the culture supernatant, washed once in PBS and fixed in 70% ethanol for cell cycle analysis.

To inhibit Myc expression we also used a siRNA technology. The siRNA used were purchased from Qiagen: Hs_MYC_5 (#SI00300902), Hs_MYC_7 (#SI02662611), Hs_MYC_9 (#SI03101847), Hs_LOC731404_4 (#SI03528896) targeting different areas of *MYC* mRNA and AllStars (#1027280), a nonsilencing siRNA with no homology to any known mammalian gene, as negative control.

For the transfection procedure, M14 control cells were seeded in complete medium and 24 h after seeding cells were transfected with siRNA using the HiPerFect Transfection Reagent (Qiagen), according to the manufacturer's instructions. Briefly, siRNAs were incubated in serum-free medium with HiPerFect Transfection Reagent for 10 min at room temperature. Subsequently, the mixture was diluted with medium and added to each well. The final concentration of each siRNAs in each well was 25 nM.

To inhibit Myc transcriptional activity we used the Max(77-85) peptide previously validated [31] and synthesized by Cambridge Peptides (Birmingham, UK). Twenty-four hours after seeding Max(77-85) was added to the M14 control cells at the concentration of 25 µM, giving this dose every 12 h. After 36 h of exposure to the peptide cells were treated with PTX 30 nM for 24 h in the presence of the peptide.

### qRT-PCR

M14 control cells were seeded in complete medium and after the different treatments cells were harvested and total RNA was extracted with the SV Total RNA Isolation System (Promega), including a DNase treatment before elution from the column. RNA (0.5 µg) was reverse-transcribed for 50 min at 42°C in a 40-µL reaction containing 200 ng of random hexamers (Amersham), 5× first-strand buffer (Invitrogen), 10 mM DTT, 0.25 mM deoxynucleotides (0.25 mM each dATP, dGTP, dCTP, dTTP, Invitrogen), 400 units of M-MLV RT (Invitrogen).

Equal amount of cDNA was taken for a subsequent quantitative real-time PCR carried out using an Applied Biosystems 7900HT Fast Real-Time PCR System instrument. Each PCR reaction contained sense and antisense primers at a concentration of 200 nM and the relevant UPL probe (Universal Probe Library, Roche) at a concentration of 100 nM in a final volume of 12 µL of FastStart Universal Probe Master (ROX) Reaction Mix (Roche).

The following sense and anti-sense primers and the relevant UPL probes were used: *GAPDH* (GenBank NM_002046.3) AGCCACATCGCTCAGACA and GCCCAATACGACCAAATCC, UPL probe #60; *MYC* (GenBank NM_002467.3) TTTTTCGGGTAGTGGAAAACC and TTCCTGTTGGTGAAGCTAACG, UPL probe #75; *INFG* (GenBank NM_000619.2) GGCATTTTGAAGAATTGGAAAG and TTTGGATGCTCTGGTCATCTT, UPL probe #21.

### Apoptosis assays

Apoptosis was monitored by annexin V binding, measuring the mitochondrial membrane potential (Δϕ) and determining the activation of caspase 3.

Cells were harvested, pooled with the supernatant, washed once in PBS and processed for the different assays. Annexin V assay was carried out using the Vybrant Apoptosis assay kit #2 (Invitrogen) following the manufacturer's protocol and samples analyzed by FACS.

For the measurement of the Δϕ the JC-1 staining was used [32]. After washing in PBS, cells were incubated with JC-1 2.5 µg/ml for 20 min at room temperature, in the dark. After two washes in PBS samples were immediately analyzed by FACS. As control, we used a depolarized sample treated with ionophore Valynomicin for further 15 min after JC-1 staining.

Caspase-3 activation was analyzed by the Enz-check kit (Invitrogen) following manufacturer's kit protocol.

### Cell cycle analysis

Cell cycle analysis was performed by both propidium iodide (PI) staining and pulse-chase bromodeoxyuridine (BrdU, Sigma) incorporation as previously described [15].

For the BrdU pulse-chase experiments, a pulse of 10 µM BrdU was added to the cell culture during the last 30 min before harvesting. Afterwards, BrdU-free medium was added to the cell culture and analysis performed every 2 h for further 24 h. Anti-BrdU (BD Biosciences, Italy) and FITC-conjugated F(ab′)_2_ rabbit anti-mouse IgG (DAKO) antibodies were used to detect the BrdU.

### MPM-2 staining

Mitotic cells were identified by staining with MPM-2, a mitosis specific marker. Control, Myc^(−)^ and Myc^(+)^ cell lines were exposed for increasing times (6, 12, 18, 24, 30, 42 h) to 30 nM PTX. At each indicated time, cells were harvested, washed in PBS and fixed overnight in 70% ethanol at −20°C. Cells were then rehydrated in ice-cold PBS for 10 min and permeabilized in medium plus 20% FCS and 0.5% Tween 20 for 10 min. The samples were then incubated with anti-phospho-Ser/Thr-Pro/MPM-2 conjugated with fluorescin-5-Ex,succinimidyl ester (Upstate) in medium containing 20% FCS and 0.06% Tween 20 at room temperature for 1 h. After three washes with PBS, cells were stained overnight with 5 µg/ml PI and 75 KU/mL RNase in PBS. Samples were then analyzed by FACS.

### Accumulation of the cells in the G0/G1 phase

For the G0/G1 phase arrest the control M14 cells were treated with the antibiotic rapamycin (100 nM 24 h), which reduces cell proliferation. Treatment with rapamycin was performed giving the cells the dose before starting the PTX exposure.

### Western blot analysis

The M14 control, Myc^(−)^ and Myc^(+)^ cells were treated with PTX 30 nM for 8 h, time at which apoptosis was not yet evident in order to avoid any interference with protein expression due to cell death. Untreated and treated cells were incubated in UREA buffer (8 M Urea, 100 mM NaH_2_PO_4_, 10 mM Tris pH 8), for 30 min on ice and then briefly sonicated. Proteins were subjected to SDS–polyacrylamide gels electrophoresis. The resolved proteins were blotted overnight to a nitrocellulose membrane, and the membranes were blocked in PBS 1× containing 5% NFM (Non-Fat Milk) for at least 1 h. Blots were then incubated with the following primary anti-human antibodies, Santa Cruz Biotechnology: anti-Myc polyclonal antibody (N262), anti-Myc monoclonal antibody (9E10), anti-p-c-Myc polyclonal antibody (Thr58/Ser62), anti-p-cdc2 polyclonal antibody (Tyr15), anti-cdc25C monoclonal antibody (H-6), anti-mad2 polyclonal antibody (FL-205), anti-Cyclin B1 polyclonal antibody (H-433), anti-p55 CDC polyclonal antibody (H-175); Dako: anti-bcl-2 monoclonal antibody (clone 124); Calbiochem: anti-cdc2 polyclonal antibody (Ab1); Chemicon International: anti-GAPDH monoclonal antibody (6C5), anti-BUBR1 monoclonal antibody (8G1); Oncogene Research: anti-HSP72/73 monoclonal antibody (Ab1). The relative amount of transferred protein in a given sample was quantified by scanning Xrays films and by estimating the relative arbitrary density units using the ImageQuant software. Each sample was normalized to the relative HSP 72/73, or GAPDH content and relative protein amount was calculated by referring Myc^(−)^ and Myc^(+)^ to the control untreated sample, and each treated sample to the correspondent untreated cells.

To study the kinetics of cyclin B1 degradation in the presence of PTX we analyzed cyclin B1 levels of expression in the M14 control, Myc^(−)^ and Myc^(+)^ cells at different times of PTX exposure (0, 2, 4, 6, 8, 10, 12, 14, 16, 18, 20 h) by using the anti-Cyclin B1 polyclonal antibody (H-433; Santa Cruz Biotechnology).

### Immunofluorescence analysis and uptake of the fluorescent peptide

M14 Myc^(+)^ cells were seeded on coverglass supports in complete medium. Cells fixed with methanol were permeabilized in PBS containing 0.5% Tween 20 and blocked in 5% NFM. Myc was detected using the anti-Myc antibody 9E10 from Santa Cruz Biotechnology. Alexa Fluor 594 Goat anti-mouse was used as secondary antibodies. Antibodies were diluted in PBS containing 2% BSA and 0.1% Tween 20 and washes were carried out in PBS containing 0.3% BSA and 0.1% Tween 20 (washing buffer). Cells were then re-blocked in PBS containing 5% NFM and then incubated with the anti-α-Tubulin (DM 1A, Sigma) and with the FITC-goat anti-mouse F(ab′)_2_ (Dako) antibody. After six washes with washing buffer the nuclei were stained with 1 µg/ml DAPI for 30 sec in PBS. Finally, cells were washed in PBS, briefly rinsed in ddH_2_O and glasses were mounted in ProLong Gold anti-Fade Reagent (Molecular Probes). Images were acquired through a fluorescence microscope Olimpus BX51.

To study the localization of Myc at the spindle poles we used a confocal laser scanning microscope (TCS SP5; Leica Microsystem, Wetzlar, Germany). The spindle poles were visualized by using γ-tubulin which is widely employed as marker for microtubule organizing centers [33]. The study was performed in four different cell lines (M14, Caco2, C2C12-Myc, HeLa). The antibodies used were: anti-Myc (monoclonal 9E10, polyclonal N262 and A14, from Santa Cruz Biotechnology; monoclonal 3C7, Chemicon) and anti γ-tubulin (Santa Cruz Biotechnology). The secondary antibodies were: Alexa Fluor 594 Goat anti-mouse IgG, Alexa Fluor 555 Donkey anti-rabbit IgG and Alexa Fluor 488 Goat anti-rabbit F(ab′)_2_ fragment antibodies. In order to avoid any possible cross-talk between secondary antibodies we chose antibodies generated in Donkey, a species known to show negligible crossreactivity, or F(ab′)_2_ fragments. To exclude cross-talk between the emission spectra of fluorophores we acquired confocal images using the sequential acquisition modality. Brightness and contrast of the acquired images were adjusted, and .tiff files of separated and merged channels were exported using Leica Application Suite 6000. Figures were generated using Adobe Photoshop 7.0 and Adobe Illustrator 10.

To study the intracellular uptake and localization of the fluorescent Max(77-85) peptide, M14 control cells were seeded on μ-slides 8 well (ibidi GmbH, Germany) in complete medium. After 24 h the TRITC-conjugated Max(77-85) peptide was added for 6 h in Optimem (Gibco). The medium was then replaced by fresh peptide-free Optimem containing HOECHST 33342 (Sigma Aldrich) at the concentration of 1 µg/ml and the cells were immediately analyzed by confocal microscopy as described above.

## Results

### Myc overexpression prevents the inhibitory effect produced by PTX in M14 melanoma cell proliferation

**Figure 1:**
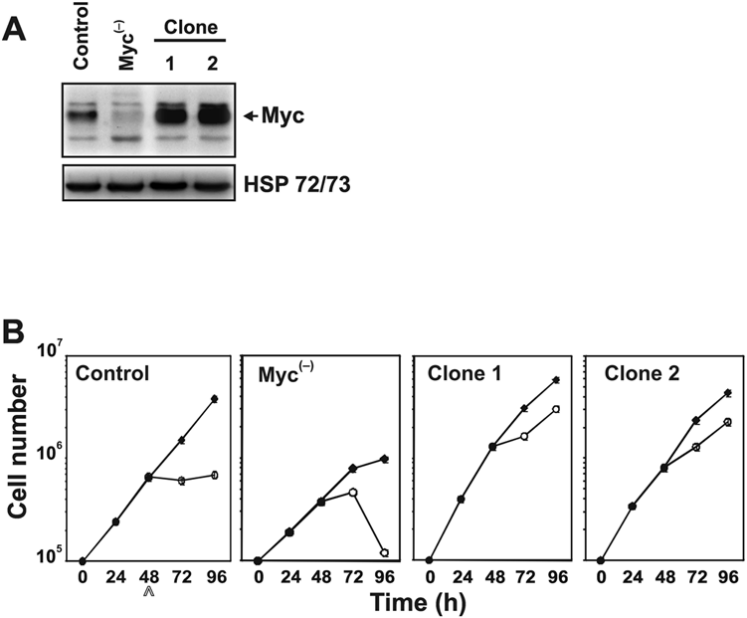
Reduced antiproliferative effect of PTX in Myc overexpressig M14 cells. (A) Levels of Myc protein in the Myc^(−)^ and Myc overexpressing clones as detected by Western blot analysis using anti-Myc N262 polyclonal antibody. Each lane was loaded with 70 µg of proteins from cell lysate. HSP 72/73 was used as a loading control. The experiment was repeated three times showing similar results. A representative blot is presented. (B) Cell growth curves of control, Myc^(−)^ and Myc overexpressing clone 1 and 2 exposed to the PTX dose of 30 nM (o—o), untreated cells (♦--♦). *Bars*, standard deviation. Arrow indicates starting of PTX treatment.

In this study we investigated the role of the *MYC* gene in the survival signalling during G2 and M phases. As death activator in such cell cycle phases we used the disruptor of microtubules PTX that is known to arrest the cells in the mitotic phase. We used two cell clones derived from the melanoma M14 cell line in which we modulated the expression of Myc protein. To reduce the levels of Myc expression, we employed the M14 pIND*c-myc* AS cell clone stably expressing a hormone/inducible *MYC* antisense RNA (named throughout this paper Myc^(−)^ cells when treated with ponasterone as indicated in Materials and Methods) that we previously described [16]. We excluded that inducing antisense RNA in M14 cells caused interferon (IFN) signalling by studying the expression level of IFNγ mRNA after ponasterone induction of the *MYC* AS RNA (Figure S1). To overexpress Myc in the same cell line, M14 cells were transfected with the pCDNA3-hMyc. G418-resistant clones were isolated after a selection period of 3 weeks and their Myc expression levels were analyzed by Western blotting (data not shown). Figure 1*A* shows Western blot analysis of two out of ten of Myc overexpressing cell clones (clones 1 and 2). These two clones show a Myc protein increment of about 3-fold. In the figure are also shown the level of expression of Myc protein in the Myc^(−)^ cells. As previously reported, 48 h of ponasterone exposure reduced the Myc level by more than 2-fold as compared to the parental M14 cell line (control cells) [16].

To choose the optimal concentration of PTX to use in our experiments we calculated the PTX IC_50_ value in control cells using four different PTX doses (1, 10, 30 and 100 nM) (data not shown). The PTX dose of 30 nM was chosen as optimal dose since represents the IC_50_ in our experimental model. Figure 1*B* shows the effect of 30 nM PTX on cell growth rate of the Myc^(−)^ and of the two Myc overexpressing clones compared to the control cell line. PTX inhibits cell proliferation in control cells by about 70% after 48 h of exposure. The PTX growth inhibitory effect was enhanced in Myc^(−)^ cells, achieving values of about 90%. On the other hand, overexpression of Myc reduced the inhibitory effect produced by PTX on cell growth with inhibition of about 40%, indicating that Myc could play a central role in the survival mechanisms of such cells. Since the two Myc overexpressing clones showed similar levels of Myc and similar response to PTX in terms of cell growth, we chose to use for further experiments only the clone 1, referred throughout this paper as Myc^(+)^ cells.

### Myc overexpression prevents apoptosis induced by microtubules damage

**Figure 2:**
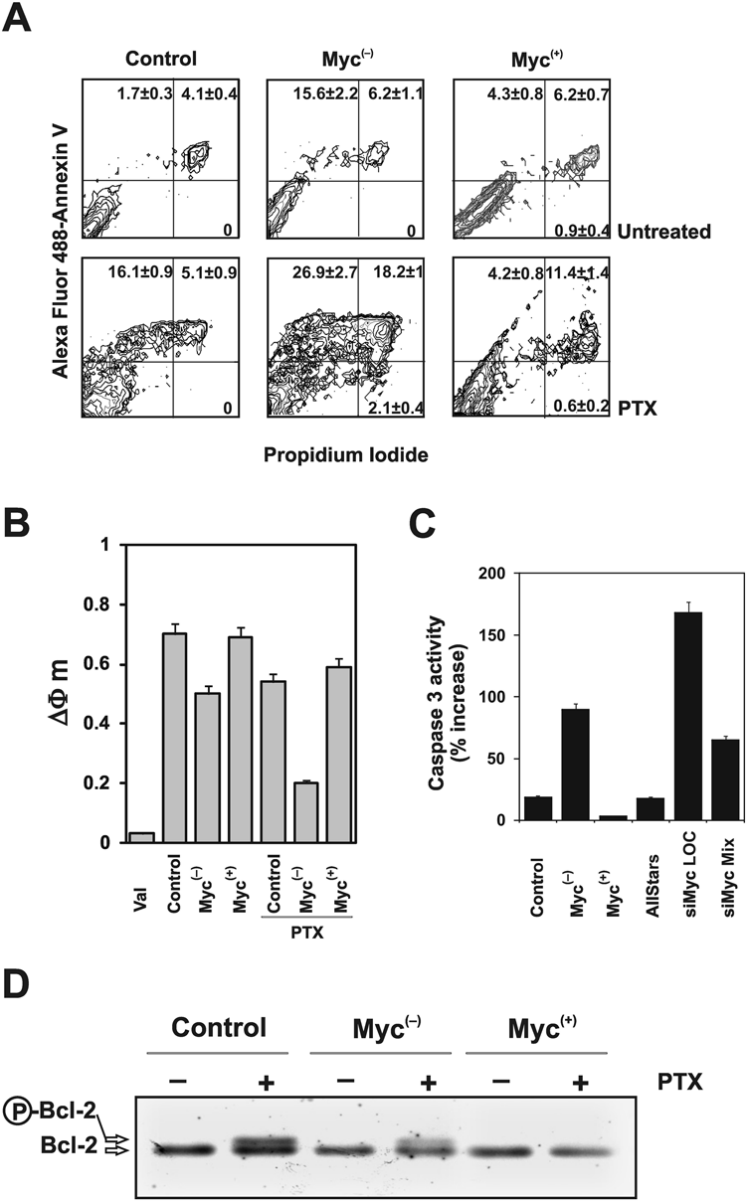
Myc overexpression prevents apoptosis in melanoma cells. (A) Detection of apoptosis by Annexin V *vs* propidium iodide assay as evaluated by flow cytometry after 24 h of PTX exposure. The mean percentages±SD reported in each cytogram represent early apoptotic (upper left), late apoptotic (upper right), necrotic (low right) and living (low left) cells. The cytograms reported are representative of three different experiments with similar results. (B) Mitochondrial membrane potential analyzed by JC-1 staining. ΔΦm in untreated and PTX treated cells are shown. Valinomycin (Val) was used as positive control to detect depolarized mitochondrial membrane potential. Data are average of at least three separate experiments. *Bars* represent standard deviation. (C) Caspase 3 activity evaluated by the Enz-check kit in control, Myc^(−)^, Myc^(+)^ and *MYC* silenced M14 cells after 24 h exposure to PTX. Data are reported as percent increase *vs* untreated cells and are average of three separate experiments. *Bars* represent standard deviation. (D) Western blot analysis of bcl-2 protein expression in the three cell lines after PTX treatment. Each lane was loaded with 40 µg of proteins from cell lysate. The experiment was repeated three times showing similar results. A representative blot is shown.

High level of inhibition of Myc^(−)^ cell growth produced by PTX was consistent with the marked activation of apoptosis induced by the drug. The measurement of apoptosis using Annexin V (Figure 2*A*) revealed that in control cells the disruption of microtubules by PTX induces a 21% cell death after 24 h of exposure to the drug. Apoptosis increased up to about 43% when Myc was down-regulated. Conversely, overexpression of Myc reduced the PTX-induced cell death down to about 15.5%.

Figure 2*B* shows that PTX activated the death signaling *via* mitochondria, as demonstrated by the measurement of the mitochondrial membrane potential. Untreated and PTX-treated cells were stained with the lipophilic cationic fluorescent probe JC-1, which localizes into the mitochondria and reports the mitochondrial membrane potential. The down-regulation of Myc induced *per se* a depolarization of the mitochondrial membrane, as shown by the decrease of ΔΦ_m_ (0.5 vs 0.7 in control cells), while overexpression of the oncoprotein did not alter significantly the ΔΦ_m_ (0.69 vs 0.7 in control cells). PTX treatment decreased mitochondrial membrane potential by 21% in control cells (ΔΦ_m_ = 0.54) and by 60% in Myc^(−)^ cells (ΔΦ_m_ = 0.2), indicating a synergism between PTX treatment and Myc reduction. The Myc^(+)^ cells exposed to PTX showed instead a ΔΦ_m_ very similar to the Myc^(+)^ untreated cells (ΔΦ_m_ = 0.6 vs 0.69 in untreated Myc^(+)^ cells).

The depolarization effect shown in Myc^(−)^ cells is consistent with the activation in the same cells of caspase 3 after 24 h PTX exposure (Figure 2*C*). We demonstrated the activation of caspase 3 in Myc down-regulated cells also using the siRNA methodology. M14 control cells were transfected for 24 h with four distinct siRNAs, targeting different areas of the *MYC* mRNA (siMyc5, siMyc7, siMyc9, siMyc LOC). We first verified the efficacy of such siRNAs on the expression of *MYC* mRNA as shown in Figure S2*A*. The strongest inhibition was elicited by the siMyc LOC. siMyc5 and siMyc7 showed a good inhibitory effect, while siMyc9 did not display any inhibitory activity. On the basis of the qRT-PCR results we chose, for further experiments, the siMyc LOC. To increase the efficacy of *MYC* silencing we also mixed the most effective siRNAs (siMyc Mix). In Figure S2*B* is reported the expression of *MYC* mRNA after silencing using siMyc Mix. As negative control the AllStars siRNA was employed in each experiment.

Treatment of M14 *MYC* silenced cells with PTX induced the activation of caspase 3 after 24 h of exposure (Figure 2*C*).

Since PTX treatment induces phosphorylation of the anti-apoptotic protein Bcl-2 [34], we analyzed Bcl-2 status by western blot analysis. As expected, PTX induced Bcl-2 phosphorylation in control cells. Such phosphorylation was still evident in Myc^(−)^ cells, even though the overall level of expression of Bcl-2 was markedly reduced. By contrast, in Myc^(+)^ cells the Bcl-2 was not phosphorylated after PTX treatment and only a slight decrease in its expression level was observed (Figure 2*D*).

### Myc overexpression enhances the endoreduplication primed by PTX

**Figure 3:**
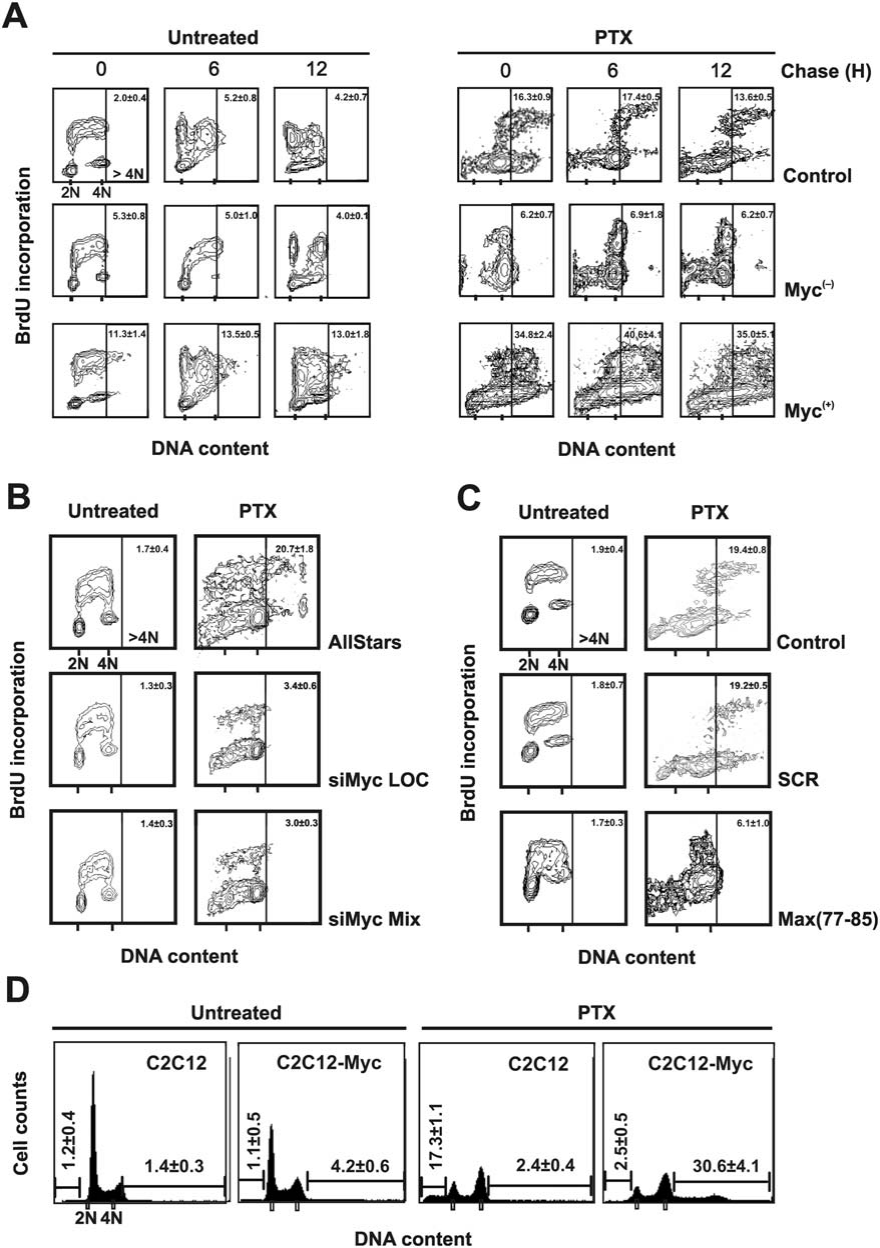
Myc overexpression enhances PTX-induced endoreduplication. (A) Flow cytometric biparametric cell cycle analysis as evaluated by a pulse-chase experiment of BrdU incorporation. Untreated or PTX-treated cells were pulse labeled with BrdU for 30 min and then maintained in BrdU-free medium for different times. Every 2 h cells were harvested and processed for the detection of BrdU in order to follow the progression of the cells through the cell cycle. In the figure are reported representative cytograms referred to the 0, 6 and 12 h of chase. The top region of the cytograms represents BrdU-positive cells. Samples not incubated with the primary anti-bromodeoxyuridine antibody were used as negative controls. The number on the right top of each histogram represent the mean percentage±SD of cells in the >4N region. The experiment was repeated three times with similar results. (B) Flow cytometric biparametric cell cycle analysis as evaluated by a pulse BrdU incorporation experiment. *MYC* mRNA was silenced using two different siRNAs in M14 cells for 24 h and then exposed to PTX for further 24 h. As control we used the AllStars siRNA (see Materials and methods for details). During the last 30 min of PTX treatment, BrdU was added to the medium. After the pulse labeling the cells were harvested and fixed in ethanol 70% for further processing. In the figure are reported representative cytograms for each treatment. The number on the right top of each histogram represent the mean percentage±SD of cells in the >4N region. The experiment was repeated three times with similar results. (C) Flow cytometric biparametric cell cycle analysis as evaluated by a pulse BrdU incorporation experiment. M14 control cells were exposed for 48 h to the Max(77-85) peptide and to the scrambled (SCR) peptide used as control. After 24 h of peptides treatment the cells were exposed to PTX for additional 24 h. During the last 30 min of treatment, BrdU was added to the medium of untreated and PTX-treated cells. After the pulse labeling the cells were harvested and fixed in ethanol 70% for further processing. In the figure are reported representative cytograms for each treatment. The number on the right top of each histogram represent the mean percentage±SD of cells in the >4N region. The experiment was repeated three times with similar results. (D) DNA content profiles in C2C12 and C2C12-Myc cell lines treated or not with PTX. The numbers reported on the left and on the right of each histograms represents the mean percentage±SD of hypodiploid and of >4N cells, respectively. The experiment was repeated three times with similar results.

To investigate the mechanism underlying the activation of apoptosis during the G2 and M phases in Myc^(−)^ cells we studied distribution of control, Myc^(−)^ and Myc^(+)^ cells in the different cell cycle compartments after exposure to PTX 30 nM for 24 h. PTX arrested about 50% of the control cells in the G2/M phases. The inhibition of Myc expression reduced the percentage of M14 cells arrested in G2/M to 27%; while, overexpression of Myc resulted in a more evident arrest in G2/M after PTX (62%, data not shown). The Myc^(−)^ cells, consistent with the reduced G2/M accumulation after PTX treatment, showed a reduced cell percentage in the >4N region (2% vs 14% in control cells, data not shown). These data indicate that the higher activation of apoptosis following PTX treatment in Myc^(−)^ cells could be associated with the prevention of PTX-induced endoreduplication. PTX produces a non-complete arrest in mitosis. The cells which adapt to the PTX-induced damage can escape this block entering in a post-mitotic status G1-like [35], with an interphase morphology and the ability to replicate, and hence duplicate DNA (endoreduplication) reaching a DNA content >4N. To clarify this point we performed a bromodeoxyuridine (BrdU) pulse-chase experiment with the three cell lines treated or not with PTX. After PTX treatment, cells were exposed to BrdU for a pulse of 30 min. BrdU incorporation was monitored immediately at the end of BrdU exposure and after 6 and 12 h. Figure 3*A* shows that down-regulation of Myc prevented PTX-induced endoreduplication compared to control cells. This effect was evident at the end of the PTX treatment (coincident with the end of the BrdU incorporation) and was maintained for subsequent 6 and 12 h, suggesting it was irreversible. By contrast, Myc^(+)^ cells exhibited a higher percentage of >4N cells compared to control cells.

To confirm that the down-regulation of Myc prevented PTX-induced endoreduplication we also performed a BrdU incorporation experiment after silencing of the *MYC* mRNA in M14 cells. Figure 3*B* demonstrates that two different siRNA *MYC* silencing were able to block PTX-induced endoreduplication.

To demonstrate further that the inhibition of Myc avoids PTX-induced endoreduplication, we blocked Myc transcriptional activity using Max(77-85), as previously reported [31]. This peptide interferes with Myc-Max heterodimerization, resulting in impaired Myc transcriptional activity. A 48 h treatment of control cells with the Max(77-85) peptide significantly inhibited the transcription of two Myc target genes, nucleolin and ODC1, without affecting expression level of Myc protein (data not shown). Figure 3*C* shows that inhibition of the Myc-Max heterodimerization prevented PTX-induced endoreduplication. As control treatment we used a scrambled peptide sequence (see [31]), which did not elicit any effect on the endoreplicative process primed by PTX exposure. In conclusion, these data suggested that the Myc-induced endoreduplication was dependent on Myc-Max heterodimerization.

Next we investigated the endoreduplication process using the non-tumoral mouse C2C12 cells (displaying barely detectable levels of Myc) and C2C12-Myc, overexpressing the Myc protein [30]. Figure 3*D* shows that, the PTX treatment elicited a relevant percentage of endoreduplication in C2C12-Myc cells (about 30%) as compared to parental C2C12 (about 2%). The PTX-induced apoptosis was also significantly higher in the C2C12 cells (about 17%) compared to the C2C12-Myc cells (about 2%).

**Figure 4:**
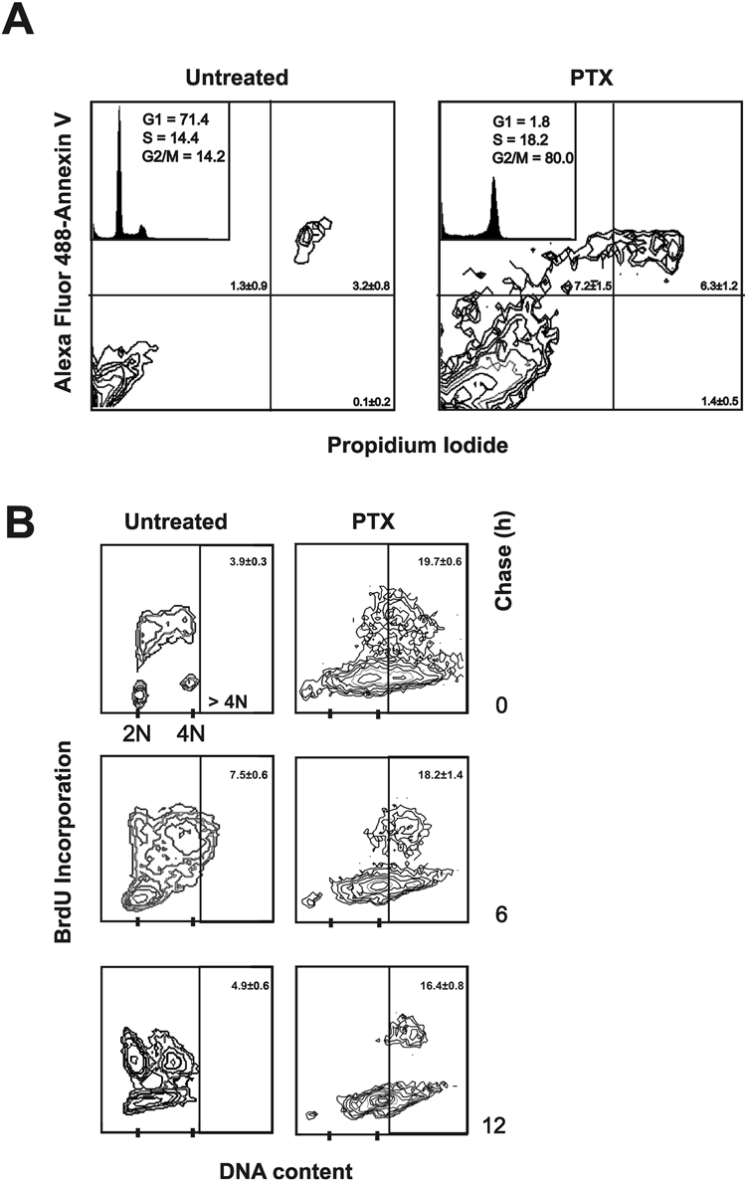
Accumulation of M14 cells in the G0/G1 cell cycle phase does not increase PTX-activated apoptosis. To accumulate cells in the G0/G1 phase we used the antibiotic rapamycin, known to induce G0/G1 arrest. (A) Apoptosis was analyzed by annexin V assay in control cells accumulated in G0/G1 phase by rapamycin and then exposed to PTX. The cytograms are representative of three independent experiments showing similar results. The number in each quadrant of the cytograms represents the mean percentage±SD of the cells in such compartment. The inserts show the relative DNA content histograms with the cell cycle percentages estimated by a mathematical model. (B) Pulse chase experiment after BrdU incorporation performed as in figure 3, in rapamycin-G0/G1-accumulated cells exposed to PTX. In the figure are reported representative cytograms referred to the 0, 6 and 12 h of chase. The number on the right top of each histogram represents the mean percentage±SD of cells in the >4N region. Experiments were repeated three times with similar results.

To exclude the possibility that the apoptotic threshold could be lowered by the G0/G1 block produced by Myc down-regulation, we examined activation of the apoptotic pathway in control cells arrested in G0/G1 phase. To accumulate the cells in G0/G1 we used the antibiotic rapamycin which is known to arrest cells in this cell cycle compartment. Rapamycin, even accumulating the cells in the G0/G1 phase (71.4%), did not increase the level of apoptosis induced by PTX (Figure 4*A*). Interestingly, apoptosis was slightly decreased in rapamycin-treated cells (about 13%) compared to control cells (about 21% – see Figure 2*A*). Furthermore, endoreduplication was not inhibited by the rapamycin-induced G0/G1 block, further supporting the hypothesis that the down-regulation of Myc may be directly responsible of the inhibition of PTX-induced endoreduplication (Figure 4*B*).

### Myc down-regulation prevents G2→M transition

**Figure 5:**
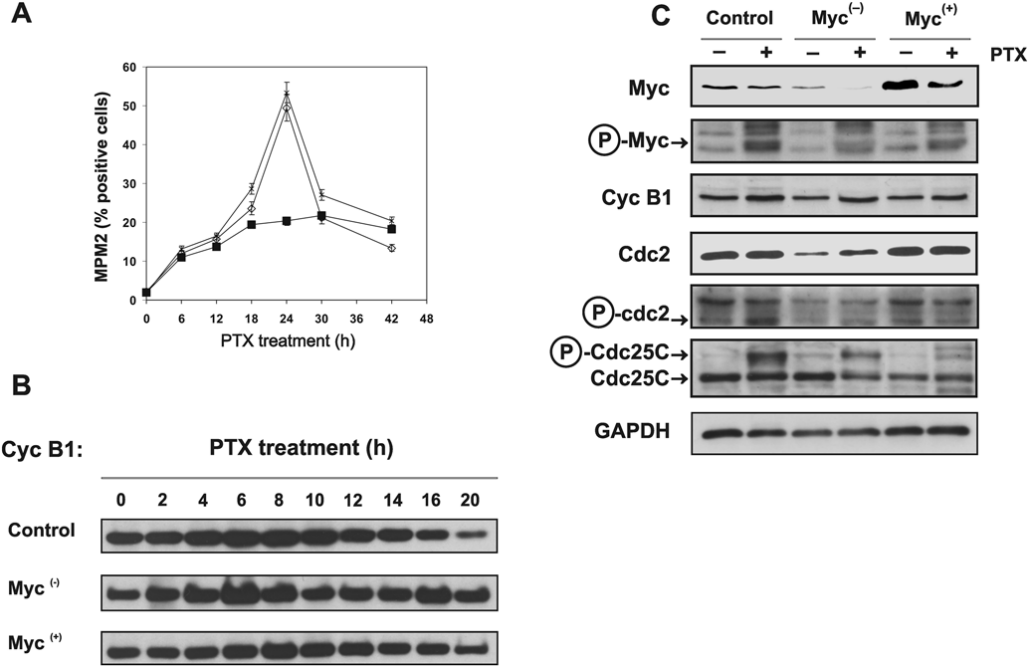
Myc down-regulation prevents cell entry into M phase. (A) Mitotic index as estimated by MPM2 positivity evaluated by flow cytometry. Cells were exposed to PTX for the indicated times and then harvested and processed for MPM2 immunofluorescence. Samples not incubated with the primary anti-MPM-2 antibody were used as negative controls. Percentages of MPM-2 positive cells were estimated by setting a marker on the control untreated cells in each MPM-2/DNA content cytogram and calculated by the method of 2–5% of background. Data are average of three different experiments with similar results (*bars*, SD). ◊ = control, * = Myc^(+)^, ▪ = Myc^(−)^. (B) Western blot analysis of cyclin B1 in the three M14 cell lines at the indicated time after PTX exposure. Each lane was loaded with 40 µg of proteins from cell lysates. The experiment was repeated three times showing similar results. A representative blot for each protein is reported. (C) Western blot analysis of the indicated protein in the three cell lines with or without 8 h exposure to PTX. Each lane was loaded with 80 µg of proteins from cell lysates. The relative amount of transferred protein in a given sample was quantified by estimating the relative arbitrary density units normalized to GAPDH content. In this blot Myc protein was detected by the monoclonal antibody 9E10. The experiment was repeated three times showing similar results. A representative blot for each protein is reported.

To measure cell entry from G2 phase into mitosis we used MPM2 staining and FACS analysis to calculate the mitotic index. The anti-MPM2 antibodies are a valuable tool for studying regulation of mitotic events since it recognises a subset of mitosis-specific phosphoproteins. Figure 5*A* shows that control and Myc^(+)^ cells transiently synchronized in M phase, 24 h after treatment with PTX. After this transient mitotic delay, both control and Myc^(+)^ cells exited mitosis, as revealed by their decreased positivity for the MPM2 antigen. The differences in the kinetics of mitotic slippage between control and Myc^(+)^ cells are more evident when the values of MPM2 positivity were normalized to the percentages of apoptotic cells measured in each sample (Figure S3). On the contrary, only few Myc^(−)^ cells showed a very slight increase of positivity for MPM2 antigen up to 42 h of PTX treatment. It is likely that the inhibition of Myc expression prevented these cells to enter the M phase thus arresting them in the G2 phase. These results are consistent with the block of endoreduplication observed in BrdU pulse-chase experiments (see. Figure 3).

To further support these results we analyzed protein levels of cyclin B1 as a measure of the G2→M transition in the presence of PTX. In Figure 5B are reported the western blot analysis in control, Myc^(−)^ and Myc^(+)^ cells of cyclin B1 evaluated at the indicated time from PTX exposure. Consistent with the very low mitotic index is the stabilization of cyclin B1 up to 20 h of PTX exposure in the Myc down-regulated cells demonstrating that the absence of Myc protein prevents the transition of the cells from the G2 to the M phase. By contrast, both control and Myc^(+)^ cells did not show any relevant differences in the kinetics of cyclin B1 degradation up to 20 h of PTX exposure.

Next we examined in the three cell lines, with or without 24 h treatment with PTX, the expression levels and phosphorylation status of a number of proteins involved in the regulation of the G2 checkpoint (Figure 5*C*). The amount of Myc was distinctive of each cell line, as expected, and decreased following PTX treatment. PTX induced an increase of the phosphorylated Myc. All the lane of p-Myc were normalized for the total amount of the Myc protein in each cell line. Then the phosphorylation in all PTX-treated cells were compared to the control treated cells. Myc^(−)^ cells show a higher level of Myc phosphorylation than the control cells and this is associated with absence of endoreduplication (see Figure 3). Conversely, in the Myc^(+)^ cells, showing a higher level of endoreduplication, the amount of Myc present in PTX-treated cells is scarcely phosphorylated. We consider the level of Myc phosphorylation inversely proportional to the ability of the cells to endoreduplicate, bypassing the mitotic PTX-induced arrest.

The level of cyclin B1 increased in all the three cell lines after PTX exposure. The amount of cdc2 decreased in Myc^(−)^ cells and instead did not change in Myc^(+)^ cells compared to control cells. cdc2 level in Myc^(−)^ cells was increased about 2-fold in PTX treatment, while it was not affected in control and Myc^(+)^ cells. The phosphorylation status of cdc2 in control cells increased 1.5-fold after PTX treatment, indicating a reduction of the kinase activity induced by microtubule damage. By contrast, in Myc^(+)^ cells cdc2 did not appear to be phosphorylated after PTX treatment, supporting the hypothesis that Myc overexpression may allow bypassing the PTX-mediated arrest in mitosis, thus increasing the amount of endoreduplication. After PTX treatment, in the control and Myc^(−)^ cells the phosphorylation status of the mitosis-inducing phosphatase Cdc25C and of cdc2 increased together, while in the Myc^(+)^ cells the very low level of cdc25C phosphorylation correspond to a very low cdc2 phosphorylation.

A quantitative densitometric analysis of the amount of the specific protein analyzed in each sample was performed for each blot and the results are reported in the Figure S4A.

### Myc localizes at the spindle poles in prometaphase

**Figure 6:**
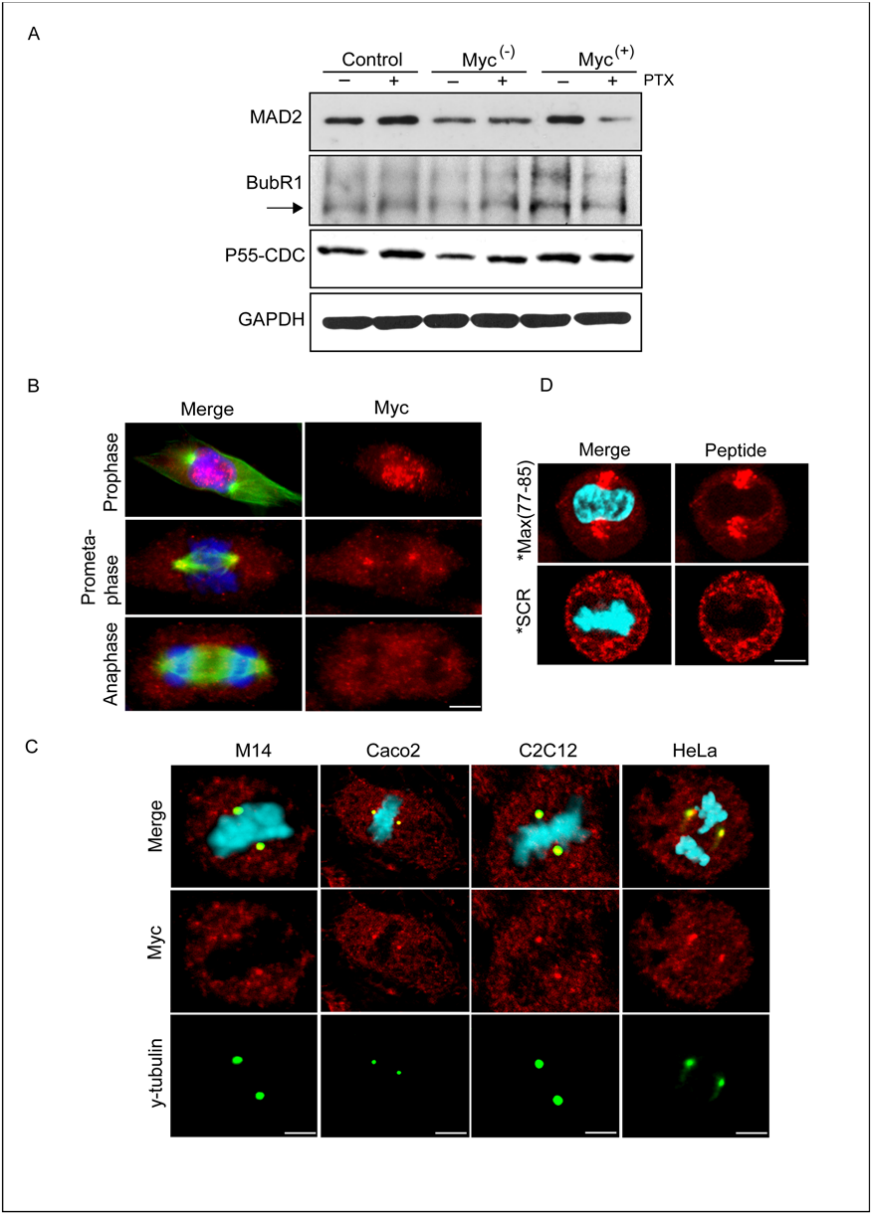
Myc localizes at the spindle poles in prometaphase. (A) Western blot analysis of the indicated protein in the three cell lines with or without 24 h exposure to PTX. Each lane was loaded with 80 µg of proteins from cell lysates. The relative amount of transferred protein in a given sample was quantified by estimating the relative arbitrary density units normalized to GAPDH content. The experiment was repeated three times showing similar results. A representative blot for each protein is reported. (B) M14 Myc^(+)^ cells in prophase, prometaphase and anaphase immunostained with anti-Myc and anti α-tubulin antibodies and with the DNA dye Dapi (scale bar 4 µm). In the prometaphase is evident the formation of a bipolar metaphase spindle. Immunofluorescent images localize Myc to the spindle poles (Merge: Myc, red; α-tubulin, green; DNA, blue). (C) Representative confocal images of spindles after immunostaining with anti-Myc clone 9E10 and anti γ-tubulin antibodies in M14 (scale bar 3 µm), Caco2 (scale bar 12 µm), C2C12-Myc (scale bar 4 µm) and HeLa (scale bar 5 µm) cells (Merge: Myc, red; γ-tubulin, green; DNA, blue). (D) Representative confocal images of the intracellular distribution of TRITC-conjugated Max(77-85) peptide (TRITC indicated with *) in M14 control cells during the prometaphase (Merge: TRITC-conjugated Max(77-85), red; DNA, blue; scale bar 4 µm).

To investigate the status of the mitotic spindle assembly checkpoint (SAC) we initially studied the expression level of some protein involved in the regulation of this checkpoint. In Figure 6*A* is reported the western blotting of three proteins which monitor the SAC. Expression level of the MAD2 protein follows the levels of Myc, increasing when Myc is overexpressed and decreasing, instead, with the Myc down-regulation, confirming that Myc regulates the transcription of MAD2. Following PTX treatment MAD2 increased in control cells and slight increased in Myc^(−)^ cells, indicating that it may be necessary for the PTX-induced activation of the SAC. Conversely, MAD2 decreased significatively in the Myc^(+)^ cells treated with PTX, suggesting that the overexpression of Myc may weaken the mitotic checkpoint. As for MAD2 the expression level of BubR1 decreases in Myc down-regulated cells, while it increases when Myc is up-regulated. Conversely, it does not significantly change after PTX in all the three cell lines. p55 CDC, homologous of the yeast Cdc20, was found decreased in the Myc down-regulated cells. A statistical densitometric analysis for each blot presented in Figure 5C is reported in the Figure S4*B*.

To go insight the involvement of Myc in the SAC we studied the Myc intracellular localization during the mitotic process. Figure 6*B* shows Myc protein localization in Myc^(+)^ cells at three subsequent phases during mitosis. During the prophase Myc immunofluorescence appeared distributed in subnuclear domains in a number of intensely fluorescent spots which tended to aggregate, during the subsequent prometaphase the immunofluorescence intensity decreased throughout the nucleus with the exception of the spindle poles, and finally faded away during the anaphase. Figure 6*C* show the selective localization of Myc at the centrosome during the prometaphase in different cell lines (M14, Caco2, C2C12-Myc and HeLa). We verified that co-localization of Myc with γ-tubulin occurred only during the prometaphase (data not shown). The prometaphase-specific localization of Myc at the centrosome suggests a direct role of Myc in the mechanism controlling the endoreduplication. The same prometaphase-specific localization of Myc was observed also, even if more faintly, in the M14 control and Myc^(−)^ cells (data not shown).

The prometaphase specific localization of Myc was visible using both the 9E10 (see Figure 6*B and C*) and the A14 (data not shown) antibodies, but not the N262 and the 3C7 antibodies (Figure S5). Since the 9E10 and A14 antibodies both recognized the C-terminal domain of the Myc protein while the N262 and the 3C7 are directed versus the N-terminus of the protein, the most feasible explanation is that Myc may interacts through its N-terminus with an unknown protein of the centrosome. This hypothesis is explained in the scheme of the Myc protein reported in Figure S6.

Next we examined the prometaphase-specific Myc localization by using a non-immunologic technique. For this purpose we studied the intracellular distribution of TRITC-conjugated Max(77-85) and SCR peptides, described above [31]. Control cells were incubated for 6 h with the fluorescent peptides and immediately analyzed by confocal microscopy. Figure 6*D* shows the intracellular distribution of the Max(77-85) and the SCR fluorescent peptides. The Max(77-85) peptide, which specifically binds Myc protein, during the prometaphase was visibly concentrated at a position which strongly reminded the two spindle poles, while the control fluorescent SCR signal was diffuse in the cytoplasm.

## Discussion

In this study we demonstrate that the activity of Myc reduces the PTX-induced apoptosis and enhances the endoreduplication primed by the disruption of microtubules. We have used three different strategies to modulate negatively Myc activity in our cellular model, the melanoma derived M14 cell line. First we used a cell clone in which the expression of Myc is down-regulated by an inducible *MYC* anti-sense RNA using the *Drosophila* hormone ecdysone [16]. Secondly we silenced *MYC* mRNA in M14 cells by using siRNA directed to *MYC* mRNA. The third approach was to inhibit Myc transcriptional activity with the Max(77-85) peptide that blocks Myc-Max heterodimerization reducing the activity of promoters activated by Myc and decreasing the expression of Myc gene targets [31]. Inhibition of Myc transcriptional activity using these peptides without significantly altering the expression level of the oncoprotein, allowed us to separate effects of Myc that are not dependent on its transcriptional activity.

To simulate a Myc overexpression status, we selected M14 clones that stably express 3-fold higher level of Myc protein than the parental cell line.

Here we have focused on the role of *MYC* gene in the survival signaling during the G2 and M phases. To activate apoptosis in these phases of cell cycle, we used PTX, a drug that induces suppression of spindle microtubule dynamics and inhibits mitosis occurrence [35]. We show that Myc overexpression protects cells from PTX-induced apoptosis and enhances endoreduplication primed by PTX. We have previously reported that the down-regulation of Myc in melanoma cells is able to activate apoptosis [16] and to increase the apoptotic response to a variety of anticancer treatments including cis-platin [36], γ-irradiation [15], melphalan [17], thus enhancing their antitumoral effect. Also Chow and colleagues [13] reported that the down-regulation of Myc determines the sensitivity of acute myeloid leukemia to the 2-Methoxyestradiol which displays antitumoral activity. Our data are also in agreement with Zhang and colleagues [11] who demonstrated that Myc down-regulation sensitize medulloblastoma cells to resveratrol-induced apoptosis. On the other hand, Evan and his colleagues have repeatedly demonstrated that *MYC* gene overexpression enhances apoptosis upon survival factors deprivation [5], [6], [9]. Also Sheen et al. [37] demonstrated that ectopic overexpression of *MYC* sensitizes normal human mammary epithelial cells to γ-irradiation-induced apoptosis, even though they observed an attenuation of the mitotic checkpoint. It is important to note that substantial differences exist between the cellular model utilized by Sheen and colleagues and the melanoma model used by us as in our melanoma model, p53 is mutated [15]. Since it has been demonstrated that *MYC* gene is negatively regulated by p53 [38], [39] we deliberately chose a p53-mutated cell line in order to study the effective activity of Myc on cell survival avoiding the involvement of the tumor suppressor protein, p53 that is widely known to activate apoptotic cell death. Adhikary and Eilers [2] reviewed two main distinct pathways through which Myc can induce apoptosis either in a p53-dependent or -independent manner. The first consists in Myc induction of the expression of p19ARF which stabilizes p53 [40], [41], and the second in the promotion by Myc of the cytochrome C release from mitochondria in a p53-independent manner [42]. The latter pathway is partly mediated by the BH3-only protein Bim, which is also induced by Myc [43]. However, both p19ARF and Bim are not direct target of Myc and their mechanism of activation has not completely clarified [44]. On the other hand, an exogenous expression of Myc promotes proliferative activity of the cells and confers them a survival advantage. As support to this conclusion is the measurement of the cell cycle phase's length of the three cell lines, obtained by BrdU pulse-chase experiments: Myc^(+)^ cells show a shorter cell cycle length (13 h) than the control (16 h), while the Myc^(−)^ cells have a very prolonged cell cycle duration (26 h) (data not shown).

We also show that treatment with rapamycin, which is known to arrest cells in G0/G1 did not increase apoptosis after PTX exposure. It may have rather a preventive effect demonstrating that is not the arrest in G0/G1 which favors the activation of apoptosis. These findings further support the hypothesis that the inhibition of Myc protein may be directly responsible for the enhancement of PTX-induced apoptosis.

The cellular response to PTX includes besides cell death another remarkable phenotype, which is the DNA endoreduplication of arrested mitotic cells. Our results support the idea that the overexpression of Myc may cause a weakening of the spindle checkpoint, explaining the marked increase in endoreduplication observed in Myc^(+)^ cells exposed to PTX. We hypothesized that Myc down-regulation could allow restoring the mitotic checkpoint preventing aberrant mitoses. Indeed, either the decrease of Myc expression or the inhibition of Myc-Max heterodimerization impairs PTX-induced endoreduplication. This is in agreement with Zanet and colleagues [45], who demonstrated that the suppression of Myc activity in epidermis impaired the keratinocytes endoreduplication required for the cell enlargement that occurs during normal post-mitotic differentiation.

Many tumor cells have only partially functional cell cycle checkpoints, which may influence the cytotoxic effect of antitumor drugs. This effect may be either p53 dependent or p53 independent [46]. In tumor cells that frequently have nonfunctional p53, DNA- or microtubules-damage principally leads to cell cycle arrest in the M phase due to prevention of the activation of the master mitotic kinase Cdk1/Cdc2 [47], [48]. Cells are effectively arrested in prometaphase and a molecular device, the spindle assembly checkpoint (SAC), whose components localize at kinetochores, function as a “proofreading network” by creating a diffusible signal indispensable to delay anaphase progression thus ensuring the prevention of aberrant mitosis. The diffusible signal operated by the SAC consists of two main molecules, BubR1 (costitutively bound to Bub3) and MAD2 that together forms the mitotic checkpoint complex, the critical effector of the SAC. This complex binds and inhibits another protein, p55 CDC (eukaryotic homologue of the yeast cdc20). p55 CDC is the essential activator of the anaphase-promoting complex (APC), an E3-ubiquitin ligase, that finally targets, for degradation by the proteasome, cyclin B and other proteins that need to be degraded at the right time to allow the cells to proceed to anaphase [49]. Our data show that the M14 control cells possess a functioning SAC since MAD2 level increases after PTX treatment and only few cells escape the mitotic arrest produced by the PTX activity. According to data reported by Menssen and colleagues [50] who demonstrated that MAD2 is transcriptionally regulated by Myc, in our model MAD2 expression levels increased when Myc was up-regulated, conversely a reduced expression of MAD2 was obtained in Myc^(−)^ cells. Consistently with the hypothesis that the up-regulation of Myc could interfere with the SAC function, MAD2 expression decreased in Myc^(+)^ cells exposed to PTX. Wassmann and colleagues have reported that MAD2 protein is highly phosphorylated when cells escape from nocodazole-induced checkpoint arrest reducing its level of expression [51]. The decreased expression of MAD2 found in Myc^(+)^ cells after PTX treatment could be ascribed to the MAD2 phosphorylation which occurs in the cells which escape PTX-induced checkpoint arrest. On the other hand, MAD2 level does not significantly change in Myc^(−)^ cells after PTX exposure. This is likely due to the arrest of the cells in the G2 phase, which probably prevents the activation of the SAC. This conclusion is supported by the analysis of the mitotic index evaluated by MPM2 FACS positivity and by the kinetics of cyclin B1 degradation during PTX exposure; in fact Myc^(−)^ cells show a low mitotic index after PTX exposure, which instead is higher in control and Myc^(+)^ cells. These data are consistent with stabilization of cyclin B1 during PTX exposure, further demonstrating that the Myc^(−)^ cells do not underwent the mitotic slippage which occurs during PTX treatment.

**Figure 7:**
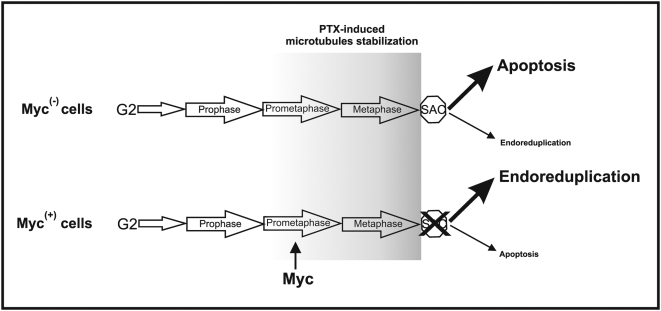
Model of the hypothetical role of Myc in the survival signaling of cells after microtubules stabilization induced by PTX in G2 and M phases. Overexpression of Myc impairs the mitotic checkpoint, promoting endoreduplication. We hypothesize that this activity displayed by Myc is manifested by the localization of Myc at spindle poles during the prometaphase. Conversely, the Myc down-regulated cells (Myc^(−)^), after PTX treatment, are blocked by the checkpoint and undergo apoptosis preventing endoreduplication.

Importantly we show for the first time that Myc localizes at the spindle poles during the prometaphase. This selective localization have been observed in four different tissue-derived cell lines implying that spindle poles localization is a conserved mechanism. The selective localization in the prometaphase stage confirmed in four different cell lines strongly suggest the possibility of a functional binding to an unknown protein. In addition, this localization is only detected using antibodies that recognize the C-terminal domain of Myc protein, indicating that its binding to the spindle pole structures occurs through the N-terminal domain to a still unidentified protein. One possibility is that Myc could bind the microtubules and the α-tubulin. Interestingly, Alexandrova and colleagues [29] have demonstrated that the N-terminal domain of Myc associates with the α-tubulin molecule and microtubules. It is known that the Myc-α-tubulin interaction is disrupted during mitosis by a specific phosphorylation of Myc at Thr-58, suggesting that the loss of such interaction at mitosis may be a physiological requirement for cell division [52]. We hypothesize that the presence of Myc at the centrosomes during the prometaphase could be necessary for the Myc-mediated attenuation of the SAC and the subsequent induction of endoreduplication. This hypothesis is schematized in Figure 7.

In addition, our data strongly suggest that the use of taxane in antitumor therapeutic strategies should be rationally based on the molecular profile of the individual tumor by specifically analyzing Myc expression levels.

## Supporting Information

Figure S1 *MYC* antisense RNA does not induce INFγ expression. Quantitative RT-PCR analysis of IFNγ mRNA levels in the control, Myc^(−)^, after induction of *MYC* AS RNA, and Myc overexpressing cells. The amounts of IFNγ mRNA were normalized to the GAPDH housekeeping gene. Data are reported as mRNA quantification relative to control cells and are average of three separate experiments. *Bars* represent standard deviation.
(0.61 MB TIF)

Figure S2 Efficacy of different siRNAs on MYC mRNA expression in M14 cells. (A) Quantitative RT-PCR analysis of *MYC* mRNA levels in the control cells transfected for 24 h with a dose of 25 nM of four distinct siRNAs (siMyc5, siMyc7, siMyc9, siMycLOC). The amounts of *MYC* mRNA were normalized to the GAPDH housekeeping gene. Data are reported as mRNA quantification relative to control cells transfected with the negative control AllStars siRNA and are average of three separate experiments. *Bars* represent standard deviation. (B) Quantitative RT-PCR analysis of *MYC* mRNA levels in the control cells transfected for 24 h with a mix of siMyc5, siMyc7 and siMycLOC (siMyc Mix), at the total dose of 25 nM. The amounts of *MYC* mRNA were normalized to the GAPDH housekeeping gene. Data are reported as mRNA quantification relative to control cells transfected with the negative control AllStars siRNA and are average of three separate experiments. *Bars* represent standard deviation.
(0.38 MB TIF)

Figure S3 Mitotic index values normalized on the fraction of apoptosis. The histograms show the mitotic index values measured 24 h after PTX exposure in the three cell lines normalized to the relative percentages of apoptosis (evaluated by annexin-V assay) at the same time of treatment. Data are average of three different experiments (*bars*, SD).
(0.43 MB TIF)

Figure S4 Densitometric analysis of Western Blots. (A) The histograms show the relative quantification analysis of the indicated proteins in figure 5C by estimating the arbitrary density units normalized against the corresponding loading control content. Data are reported as relative protein amount and are average of two or three separate experiments. *Bars* represent standard deviation. 1 = Control; 2 = Control+PTX; 3 = Myc^(−)^; 4 = Myc^(−)^+PTX; 5 = Myc^(+)^; 6 = Myc^(+)^+PTX. (B) The histograms show the relative quantification analysis of the indicated proteins in figure 6A by estimating the arbitrary density units normalized against the corresponding loading control content. Data are reported as relative protein amount and are average of two or three separate experiments. Bars represent standard deviation. 1 = Control; 2 = Control+PTX; 3 = Myc^(−)^; 4 = Myc^(−)^+PTX; 5 = Myc^(+)^; 6 = Myc^(+)^+PTX.
(0.02 MB PDF)

Figure S5 Myc protein localization. Immunofluorecence images of HeLa cells immunostained with anti-myc antibodies recognizing the N-terminal domain of Myc protein. Merged: Myc, red; γ-tubulin, Green; DNA, blue (scale bar 4 µm).
(0.69 MB TIF)

Figure S6 Scheme of Myc domains. The scheme reported draws the exact epitopes of each anti-Myc antibody (monoclonal 9E10 and polyclonal A14 directed versus the C-terminal domain; polyclonal N262 and monoclonal 3C7, directed versus the N-terminal domain) used in the immunofluorescence experiments. The numbers represent the sequence position of aminoacids. Myc-Box domains are: I, II, III. BR, Basic Region. HLH, Helix-Loop-Helix. LZ, Leucine Zipper.
(0.41 MB TIF)

## References

[1] Amati B, Alevizopoulos K, Vlach J (1998). Myc and the cell cycle.. Front Biosci.

[2] Adhikary S, Eilers M (2005). Transcriptional regulation and transformation by Myc proteins.. Nat Rev Mol Cell Biol.

[3] Hanson KD, Shichiri M, Follansbee MR, Sedivy JM (1994). Effects of c-myc expression on cell cycle progression.. Mol Cell Biol.

[4] Ryan KM, Birnie GD (1996). Myc oncogenes: the enigmatic family.. Biochem J.

[5] Pelengaris S, Khan M, Evan G (2002). c-MYC: more than just a matter of life and death.. Nat Rev Cancer.

[6] Evan GI, Wyllie AH, Gilbert CS, Littlewood TD, Land H (1992). Induction of apoptosis in fibroblasts by c-myc protein.. Cell.

[7] Nesbit CE, Tersak JM, Grove LE, Drzal A, Choi H (2000). Genetic dissection of c-myc apoptotic pathways.. Oncogene.

[8] Nilsson JA, Cleveland JL (2003). Myc pathways provoking cell suicide and cancer.. Oncogene.

[9] Juin P, Hunt A, Littlewood T, Griffiths B, Swigart LB (2002). c-Myc functionally cooperates with Bax to induce apoptosis.. Mol Cell Biol.

[10] Ayala-Torres S, Zhou F, Thompson EB (1999). Apoptosis induced by oxysterol in CEM cells is associated with negative regulation of c-myc.. Exp Cell Res.

[11] Zhang P, Li H, Wu ML, Chen XY, Kong QY (2006). c-Myc downregulation: a critical molecular event in resveratrol-induced cell cycle arrest and apoptosis of human medulloblastoma cells.. J Neurooncol.

[12] Wang YH, Liu S, Zhang G, Zhou CQ, Zhu HX (2005). Knockdown of c-Myc expression by RNAi inhibits MCF-7 breast tumor cells growth in vitro and in vivo.. Breast Cancer Res.

[13] Chow JM, Liu CR, Lin CP, Lee CN, Cheng YC (2008). Downregulation of c-Myc determines sensitivity to 2-methoxyestradiol-induced apoptosis in human acute myeloid leukemia.. Exp Hematol.

[14] Biroccio A, Benassi B, Amodei S, Gabellini C, Del Bufalo D (2001). c-Myc down-regulation increases susceptibility to cisplatin through reactive oxygen species-mediated apoptosis in M14 human melanoma cells.. Mol Pharmacol.

[15] Bucci B, D'Agnano I, Amendola D, Citti A, Raza GH (2005). Myc down-regulation sensitizes melanoma cells to radiotherapy by inhibiting MLH1 and MSH2 mismatch repair proteins.. Clin Cancer Res.

[16] D'Agnano I, Valentini A, Fornari C, Bucci B, Starace G (2001). Myc down-regulation induces apoptosis in M14 melanoma cells by increasing p27(kip1) levels.. Oncogene.

[17] Greco C, D'Agnano I, Vitelli G, Vona R, Marino M (2006). c-MYC deregulation is involved in melphalan resistance of multiple myeloma: role of PDGF-BB.. Int J Immunopathol Pharmacol.

[18] Michor F, Iwasa Y, Nowak MA (2004). Dynamics of cancer progression.. Nat Rev Cancer.

[19] Beckman RA, Loeb LA (2005). Genetic instability in cancer: theory and experiment.. Semin Cancer Biol.

[20] Wade M, Wahl GM (2006). c-Myc, genome instability, and tumorigenesis: the devil is in the details.. Curr Top Microbiol Immunol.

[21] Dominguez-Sola D, Ying CY, Grandori C, Ruggiero L, Chen B (2007). Non-transcriptional control of DNA replication by c-Myc.. Nature.

[22] Kidder BL, Yang J, Palmer S (2008). Stat3 and c-Myc genome-wide promoter occupancy in embryonic stem cells.. PLoS ONE.

[23] Wong DJ, Liu H, Ridky TW, Cassarino D, Segal E (2008). Module map of stem cell genes guides creation of epithelial cancer stem cells.. Cell Stem Cell.

[24] Prochownik EV, Li Y (2007). The ever expanding role for c-Myc in promoting genomic instability.. Cell Cycle.

[25] Felsher DW, Zetterberg A, Zhu J, Tlsty T, Bishop JM (2000). Overexpression of MYC causes p53-dependent G2 arrest of normal fibroblasts.. Proc Natl Acad Sci U S A.

[26] Cowling VH, Chandriani S, Whitfield ML, Cole MD (2006). A conserved Myc protein domain, MBIV, regulates DNA binding, apoptosis, transformation, and G2 arrest.. Mol Cell Biol.

[27] Taylor SS, Scott MI, Holland AJ (2004). The spindle checkpoint: a quality control mechanism which ensures accurate chromosome segregation.. Chromosome Res.

[28] Li Q, Dang CV (1999). c-Myc overexpression uncouples DNA replication from mitosis.. Mol Cell Biol.

[29] Alexandrova N, Niklinski J, Bliskovsky V, Otterson GA, Blake M (1995). The N-terminal domain of c-Myc associates with alpha-tubulin and microtubules in vivo and in vitro.. Mol Cell Biol.

[30] Crescenzi M, Crouch DH, Tato F (1994). Transformation by myc prevents fusion but not biochemical differentiation of C2C12 myoblasts: mechanisms of phenotypic correction in mixed culture with normal cells.. J Cell Biol.

[31] D'Agnano I, Valentini A, Gatti G, Chersi A, Felsani A (2007). Oligopeptides impairing the Myc-Max heterodimerization inhibit lung cancer cell proliferation by reducing Myc transcriptional activity.. J Cell Physiol.

[32] Salvioli S, Ardizzoni A, Franceschi C, Cossarizza A (1997). JC-1, but not DiOC6(3) or rhodamine 123, is a reliable fluorescent probe to assess delta psi changes in intact cells: implications for studies on mitochondrial functionality during apoptosis.. FEBS Lett.

[33] Job D, Valiron O, Oakley B (2003). Microtubule nucleation.. Curr Opin Cell Biol.

[34] Asnaghi L, Calastretti A, Bevilacqua A, D'Agnano I, Gatti G (2004). Bcl-2 phosphorylation and apoptosis activated by damaged microtubules require mTOR and are regulated by Akt.. Oncogene.

[35] Abal M, Andreu JM, Barasoain I (2003). Taxanes: microtubule and centrosome targets, and cell cycle dependent mechanisms of action.. Curr Cancer Drug Targets.

[36] Citro G, D'Agnano I, Leonetti C, Perini R, Bucci B (1998). c-myc antisense oligodeoxynucleotides enhance the efficacy of cisplatin in melanoma chemotherapy in vitro and in nude mice.. Cancer Res.

[37] Sheen JH, Woo JK, Dickson RB (2003). c-Myc alters the DNA damage-induced G2/M arrest in human mammary epithelial cells.. Br J Cancer.

[38] Levy N, Yonish Rouach E, Oren M, Kimchi A (1993). Complementation by wild-type p53 of interleukin-6 effects on M1 cells: induction of cell cycle exit and cooperativity with c-myc suppression.. Mol Cell Biol.

[39] Sachdeva M, Zhu S, Wu F, Wu H, Walia V (2009). p53 represses c-Myc through induction of the tumor suppressor miR-145.. Proc Natl Acad Sci U S A.

[40] Eischen CM, Weber JD, Roussel MF, Sherr CJ, Cleveland JL (1999). Disruption of the ARF-Mdm2-p53 tumor suppressor pathway in Myc-induced lymphomagenesis.. Genes Dev.

[41] Zindy F, Eischen CM, Randle DH, Kamijo T, Cleveland JL (1998). Myc signaling via the ARF tumor suppressor regulates p53-dependent apoptosis and immortalization.. Genes Dev.

[42] Juin P, Hueber AO, Littlewood T, Evan G (1999). c-Myc-induced sensitization to apoptosis is mediated through cytochrome c release.. Genes Dev.

[43] Egle A, Harris AW, Bouillet P, Cory S (2004). Bim is a suppressor of Myc-induced mouse B cell leukemia.. Proc Natl Acad Sci U S A.

[44] Baudino TA, Maclean KH, Brennan J, Parganas E, Yang C (2003). Myc-mediated proliferation and lymphomagenesis, but not apoptosis, are compromised by E2f1 loss.. Mol Cell.

[45] Zanet J, Pibre S, Jacquet C, Ramirez A, de Alboran IM (2005). Endogenous Myc controls mammalian epidermal cell size, hyperproliferation, endoreplication and stem cell amplification.. J Cell Sci.

[46] Taylor WR, Stark GR (2001). Regulation of the G2/M transition by p53.. Oncogene.

[47] Peng CY, Graves PR, Thoma RS, Wu Z, Shaw AS (1997). Mitotic and G2 checkpoint control: regulation of 14-3-3 protein binding by phosphorylation of Cdc25C on serine-216.. Science.

[48] Mullan PB, Quinn JE, Gilmore PM, McWilliams S, Andrews H (2001). BRCA1 and GADD45 mediated G2/M cell cycle arrest in response to antimicrotubule agents.. Oncogene.

[49] Murray AW (2004). Recycling the cell cycle: cyclins revisited.. Cell.

[50] Menssen A, Epanchintsev A, Lodygin D, Rezaei N, Jung P (2007). c-MYC delays prometaphase by direct transactivation of MAD2 and BubR1: identification of mechanisms underlying c-MYC-induced DNA damage and chromosomal instability.. Cell Cycle.

[51] Wassmann K, Liberal V, Benezra R (2003). Mad2 phosphorylation regulates its association with Mad1 and the APC/C.. EMBO J.

[52] Niklinski J, Claassen G, Meyers C, Gregory MA, Allegra CJ (2000). Disruption of Myc-tubulin interaction by hyperphosphorylation of c-Myc during mitosis or by constitutive hyperphosphorylation of mutant c-Myc in Burkitt's lymphoma.. Mol Cell Biol.

