# Supplementary figures and images for: Myc Prevents Apoptosis and Enhances Endoreduplication Induced by Paclitaxel
Source: PLoS One. 2009 May 6;4(5):e5442. doi: 10.1371/journal.pone.0005442 (PMC2673584; doi:10.1371/journal.pone.0005442)

**A****Relative protein amount**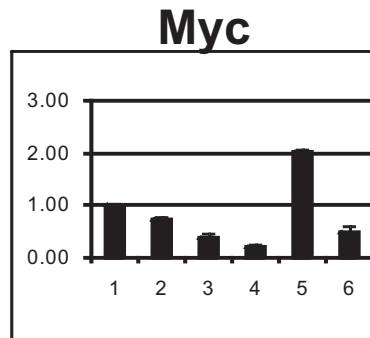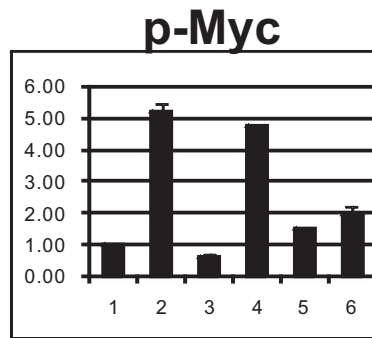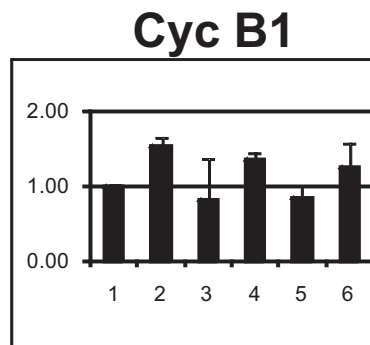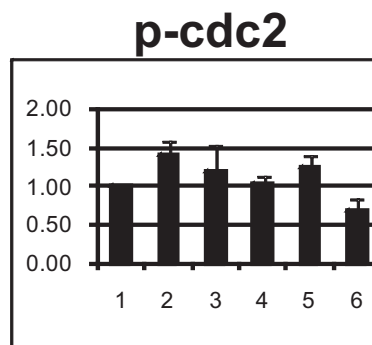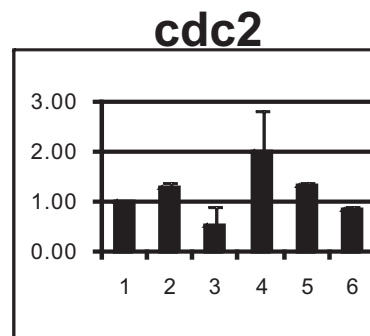**B**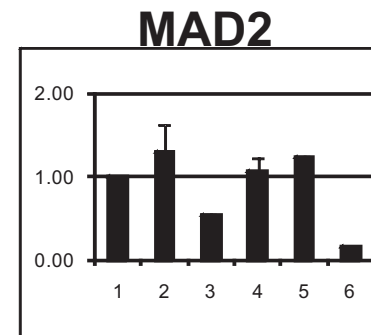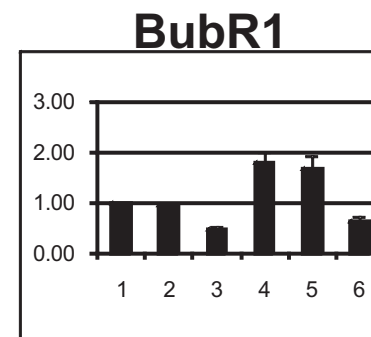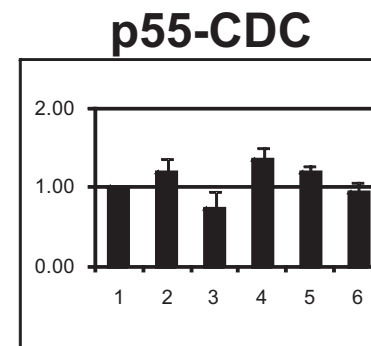

Supplement: Figure S4 — Densitometric analysis of Western Blots. (A) The histograms show the relative quantification analysis of the indicated proteins in figure 5C by estimating the arbitrary density units normalized against the corresponding loading control content. Data are reported as relative protein amount and are average of two or three separate experiments. Bars represent standard deviation. 1 = Control; 2 = Control+PTX; 3 = Myc(−); 4 = Myc(−)+PTX; 5 = Myc(+); 6 = Myc(+)+PTX. (B) The histograms show the relative quantification analysis of the indicated proteins in figure 6A by estimating the arbitrary density units normalized against the corresponding loading control content. Data are reported as relative protein amount and are average of two or three separate experiments. Bars represent standard deviation. 1 = Control; 2 = Control+PTX; 3 = Myc(−); 4 = Myc(−)+PTX; 5 = Myc(+); 6 = Myc(+)+PTX. (0.02 MB PDF) [file pone.0005442.s004.pdf]

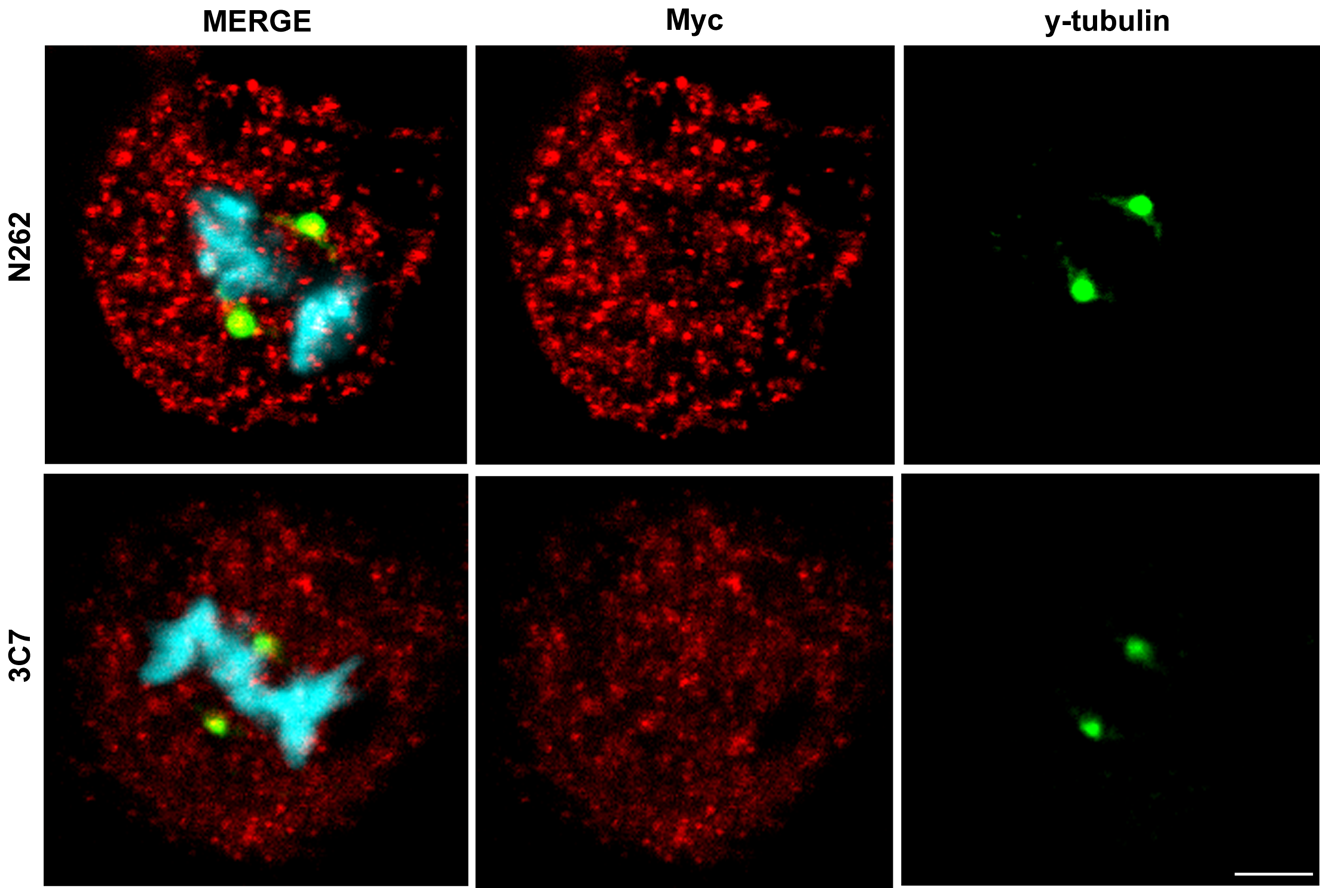

Supplement: Figure S5 — Myc protein localization. Immunofluorecence images of HeLa cells immunostained with anti-myc antibodies recognizing the N-terminal domain of Myc protein. Merged: Myc, red; γ-tubulin, Green; DNA, blue (scale bar 4 µm). (0.69 MB TIF) [file pone.0005442.s005.tif]

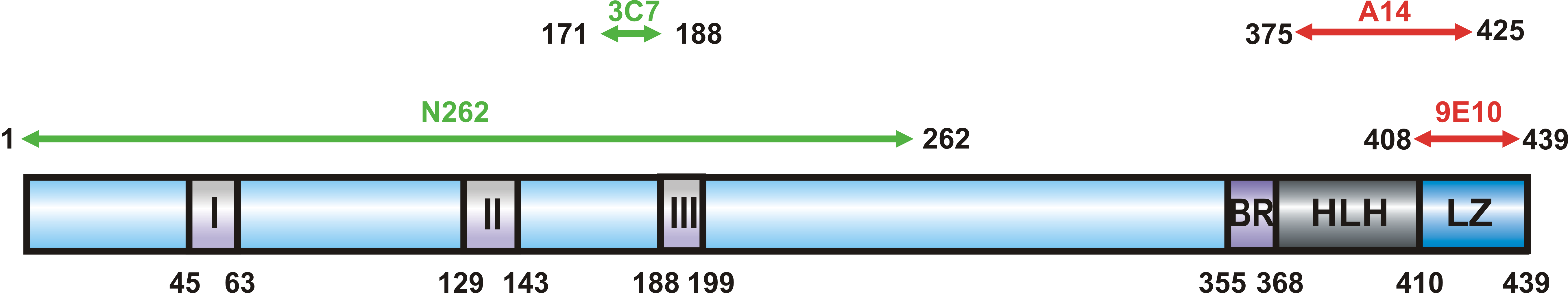

Supplement: Figure S6 — Scheme of Myc domains. The scheme reported draws the exact epitopes of each anti-Myc antibody (monoclonal 9E10 and polyclonal A14 directed versus the C-terminal domain; polyclonal N262 and monoclonal 3C7, directed versus the N-terminal domain) used in the immunofluorescence experiments. The numbers represent the sequence position of aminoacids. Myc-Box domains are: I, II, III. BR, Basic Region. HLH, Helix-Loop-Helix. LZ, Leucine Zipper. (0.41 MB TIF) [file pone.0005442.s006.tif]
